# Transformational Change and Digitalization—The Case of the Swedish Road and Transport Administration

**DOI:** 10.3390/bs13020110

**Published:** 2023-01-29

**Authors:** Fredrik Molin, Eva Norrman Brandt

**Affiliations:** 1 IPF, The Institute for Organizational and Leadership Development, Uppsala University, 753 20 Uppsala, Sweden; 2 Department of Medical Sciences, Occupational and Environmental Medicine, Uppsala University, 752 37 Uppsala, Sweden

**Keywords:** transformational change, organizational behavior, digitalization, transport administration

## Abstract

Background: Digitalization is one of the drivers of change in both public and private organizations. It is therefore relevant to understand how a government agency like the Swedish Transport Administration manage and experience change. Methods: In this qualitative study, interviews (*n* = 15) with respondents with insight and connection to digitalization and change highlight factors related to digitalization and change-capacity within the agency. Results: The results of the interviews are presented in a thematic analysis. Five themes were identified: Digitalization, management control, stability requirements, organizational culture, and lack of a comprehensive view. The research literature in the field of change creates a fund for a discussion about the Administration’s situation regarding digitalization, development, and transformational change. Conclusions: The results indicate that the Transport Administration still has a long way to travel in terms of organizational readiness for change. To address this issue, the Transport Administration should prioritize the development and implementation of a comprehensive change management strategy including clear communication, active engagement, and participation from all employees, and a focus on building a culture of adaptability and continuous improvement.

## 1. Introduction

The rate of change is increasing according to several scholars [1,2,3,4,5,6], and this increasing rate of change is driven by several factors, where technical development is a significant one. Digitalization and technical development create pressure for change and challenges old power structures and roles within organizations [2,4]. These circumstances require management, governance, and decision-making where hierarchical structures tend to become too rigid and too slow to match the demands of change and innovation [2,4,7,8,9]. Transformative change, which affects fundamental assumptions about different aspects of organizational culture, is considered particularly challenging because it challenges behaviors and fundamental principles within an organization [10,11].

Previous research shows that co-creating leaders are better equipped to lead and drive transformative change [12,13,14]. Co-creating leaders engage members of the organization in the work of change [12] and are more open to rethinking their position in exchange with both their employees and superiors [12,14,15,16].

The success-rate of change is often described as low [17,18] and the reasons why change does not reach set goals are well-known [19], but the gap between theory and practice seems to be large [20]. Previous studies have shown the role of transformative leadership, which has a strong impact on change outcomes as well as employee well-being [13]. Other research has shown a correlation between leader behavior and the success of change [15,16].

Understanding and making sense of the novelties and events in an organization is fundamental to understanding the change and transformation for members of an organization [21]. Sensemaking can be described as a first step in understanding change. In this qualitative study, the aim was to explore how employees experience change efforts focused on digitalization and change within the Swedish Transport Administration.

## 2. Theoretical Background

### 2.1. Organizational Change

A large portion of attempted change initiatives never reach their goals. In the literature, the figure of 70% often appears as the share of failed change initiatives [1,17,18,22,23]. The figure has been discussed, criticized, and problematized [17,23], but the realization that many change efforts fail has been found among both researchers and practitioners [1]. The reasons for failure are a palette of possible contributing reasons:

- Insufficient planning and inadequate “diagnosis” of the organization’s conditions [24];
- Lack of direction and support from senior management [25];
- Neglecting attention to the cultural conditions of the organizations [26];
- Lack of structure and content in change communication [27,28];
- Tensions in focus on organizational needs and the human side of change [29].

When change is considered and analyzed, it can be viewed from several different perspectives. In [30] (cited and presented in [1]), they derived the following perspectives on change: scale and scope, rate of occurrence, and how it comes about. Dunphy and Stace [31] presented a model of scope of change that has often been used as a reference to describe how extensive the change is, and what kind of leadership is needed to achieve the different degrees of change. Nadler and Nadler [32] also described different types of change challenges as part of helping leaders plan and drive different types of change efficiently.

People react to change based on many contextual conditions [33] and, moreover, people are inherently different—some are prone to change, some seek change, while others oppose it for the longest [1,34,35]. As a leader, understanding the elements of a change situation, context, content, and actors is crucial [17,33,36].

In the change literature, there are many examples of strategies to promote change [11,21,37,38] and what has emerged is that human, social, psychological, and communicative factors are important for successful organizational change [11,21]. Change theories are context-dependent, where change efforts are based on both external influences (what happens in the outside world and affects the organization) and the inner setting (values and assumptions in the specific organizational culture). Therefore, in a world with an increasing rate of change, the theories of change are not applicable to all organizations [3].

Who will represent and drive a change effort is of great importance, and champions of change need to be individuals with integrity and a strong position within the organization [39].

### 2.2. Transformative Change

In the literature, there are a number of definitions of critical changes that are called transformative. Transformative change is defined as a fundamental change involving the mindset, culture, and character of an organization [10,11,22,33,36,38]. Interest in and need for transformative change has increased in society since the 1990s [4,40]. The reasons for the greater need for transformative change are caused by technological development, innovation, global competition, and customer requirements. With increasing rates of change and disruptive events, both the ability to react and respond to external events are put to the test [4,5]. The need for changed and developed working methods becomes extra clear when many organizations need to drive innovation within its ordinary production processes [4].

In the face of transformative change, three interoperable areas need to be considered according to Pettigrew [33]: (1) The context: Why is this change being made? (2) The content: What should be changed and how can the change be measured? (3) The process: How is the change-process designed? The context consists of two parts: the external in the form of events and pressures of various kinds, and the internal in the form of the organizational culture and structure [33,36]. Clarifying the reasons for change so that the members of the organization understand the importance of acting counteracts the resistance to change that might otherwise hinder the change process [35,37,41]. The content of the change is important in measuring and demonstrating progress [33,37]. Celebrating early wins and progress increases motivation and gives an orientation as to what has already been developed and accomplished [37]. As for the process of change, the actors in the form of leaders and employees are important; leadership will play a role in a transformation in which several levels and many assumptions are challenged [14,42,43].

## 3. Materials and Methods

This qualitative study was conducted using semi-structured interviews with employees at the Swedish Transport Administration who held strategic roles related to digitalization and change work within the Administration. The interview questions focused on digitalization within the organization and its impact on individuals and groups. The answers went beyond the questions asked and included external influences and reflections on value creation, management control, and system understanding. Themes from the interviews have been identified and linked to theories of change and leadership in change.

### 3.1. Setting

The Swedish Road and Transport Administration has approximately 10,600 employees on several locations in Sweden. The mission of the Administration is to ensure the function and development of the national transport system.

The Swedish Transport Administration is divided into six central functions and six business areas as well as four profit centers. The business areas are Planning, Traffic, Maintenance, Investment, Large Projects, and ICT. Planning plans for publicly financed infrastructure for road and rail. Traffic monitors and directs traffic on roads and railways and delivers traffic information so that the system is used safely and efficiently. Maintenance manages, maintains, and develops the road and railway network and its technical systems. Investment is responsible for procurement, implementation, and follow-up of the main part of the Swedish Transport Administration’s major redevelopment measures and new investments. It manages projects that have a budget of up to SEK 1 billion. Large projects is responsible for procurement, implementation, and follow-up of the Swedish Transport Administration’s largest new investments and manages projects that have a budget of more than SEK 1 billion. Information and communication technology is responsible for the Swedish Transport Administration’s IT infrastructure and technical management of IT solutions.

### 3.2. Participants and Data Collection

In total, 15 respondents linked to change, digitalization, and strategic initiatives within the Swedish Transport Administration were interviewed. A snowball sampling strategy [44] was used for the study where the respondents recommended other respondents and key informants within the Administration. Based on such recommendations, 20 employees were contacted via email and invited to take part in the study, and 15 respondents agreed to participate. Nine male and six female respondents participated in the study. The participants held different positions within the Administration with titles such as business developer, change manager, digital strategist, project manager, department/unit manager, and enterprise architects.

The interviews took place in March–May 2020, were conducted via Skype, and the length of the interviews ranged from 60 min to 90 min. Shorthand notes were taken during the interviews and more extensive notes were written immediately after the interview. From the interview material, quotes and themes related to change, digitalization, and leadership were extracted. Quotes from the interviews were reported back to the interviewees who approved or made corrections (respondent validation/member check) [45]. The participants acknowledged the results and were able to relate them to their own context, which strengthens the credibility of the results [46].

The interviews were semi-structured [47] and covered the respondents’ views of change and digitalization within the Administration. The interview guide contained questions regarding the following areas: experiences of change within the Administration, perception of management and governance, organizational culture, and values, use and implementation of digital tools.

### 3.3. Data Analysis

The method used to analyze the interviews of the subject matter was inspired by a thematic analysis according to [48]. Analysis of the material took place in several steps. (1) Initially, the notes were read several times in order to obtain a comprehensive overview of the data material. Some of the leading ideas on possible themes emerged during this read through. In this initial phase of the analysis, all transcribed interviews were coded line by line [49]. (2) Recurrent and important phenomena were identified in the material and sentences and quotations from the interviews were added to these. (3) Important phenomena were grouped to crystallize five themes: Digitalization, Management control, Stability requirements, Organizational culture, and Lack of a comprehensive view. (4) Each theme was presented in a written account combined with quotes from the interviews.

## 4. Results

The results are presented under five main themes: Digitalization, Management control, Stability requirements, Organizational culture, and Lack of a comprehensive view.

### 4.1. Digitalization

Digitalization was experienced as a broad concept subject to different interpretations within different parts of the organization. “There are differences about what we mean by digitalization in our organization. It can be anything in IT or in technology. Knowledge of digitalization and what it means is generally low.”

The urgency of the issue seems obvious, and the respondents agreed that the highest ranks had not encouraged digital development. One problem is that the message from the highest level of management is hindered by uncertainty about what is covered by digitalization, and partly a lack of resources in the implementation of digital projects.

“My experience is that we rarely talk about change and the impact of digitalization. The risk is that we will miss the chance to have a dialogue on how we could benefit from more technology. We easily end up at an abstract level when it comes to digitalization. Digitalization is a societal change we must make.”

The pace of development is perceived as low due to the lack of an overall picture of the transformation and to local resistance. Where change support has been put in place, digitalization seems to have worked well.

“We are working on issues that are far away. Advanced AI, self-driving vehicles, electric roads, and other high-flying political goal. The Administration has business developers looking 5–10 years ahead while the line managers have a 1–2-year perspective. There is a visionary forward-looking mindset that gets stuck in the middle layer of managers. For digitalization to take off, we need to invest in creating a deeper understanding of the possibilities of digitalization.”

Line managers need to have a clear understanding of the Administration’s vision for digitalization and being able to express their own specific requirements and needs. The respondents reported that there may be a lack of understanding and a lack of involvement in ongoing digitalization efforts. Line managers and employees may not understand the benefits of digitalization and how it will affect their work, which can lead to resistance. The organization’s digitalization efforts are not always aligned with the overall strategy, which means that the specific needs of the line managers and their teams are not always met.

### 4.2. Management Control

The challenge of the large authority is for the top management level to reach out with its messages and to realize change so that it does not just become a paper product. Attempts and approaches have been made to increase collaboration within the matrix organization designed to combine the line’s work with different processes. “The Administration has appointed digital strategists with long-term and visionary goals. They are supposed to function as a kind of internal consultants.” However, the appointment of several digital strategists did not appear to be fully anchored in the business. It is an ambition of senior management, but the line managers do not really know what these roles will be used for. Additionally, the interviewees were unanimously critical of how this worked in practice. Therefore, there is a clear gap between the visionary anchors of senior management and the line managers.

Review and follow-up was also considered to be an obstacle to development, according to several interviewees. Collaboration and systems thinking are not rewarded, but rather the fulfilment of locally set goals and targets. Several interviewees expressed a frustrated view of how the interests of individual managers counteracted the direction toward collaboration that had been designed. “Top management’s ideas about digitalization are difficult to get down in the business.”

Senior management was perceived as visionary, but the bridge to the business’s everyday life was considered missing. “A new management structure would be needed to clarify which decisions should be taken centrally and which should be handled in the business areas. We need to challenge power relations and create incentives for business areas to cooperate more.”

In several interviews, shortcomings in change management skills spontaneously arose. The opportunity to collaborate with external actors who had carried out digitalization changes was mentioned in some interviews as an untapped opportunity. “We need to get better at change. There are internal examples of successful change, we could make visible and learn more from them as well as we could acquire mentoring companies that have succeeded with changes similar to those we need to make.”

### 4.3. Stability Requirements

The Transport Administration is a government authority and the demand for stability and security is great, while there is also the requirement to be a modern authority with a digitalization rate that matches society in general. “We care a lot about stability, and this can counteract the pace of digitalization, I think. And digitalization could mean more fun tasks, but we have less interest and focus on change because of the management and governance we have. There are many unused efficiencies.”

There was tension between legitimate requirements for safety and stability and the possibilities offered by new technical solutions. Arguments for stability can be fueled by examples of failures and scandals, but there is also a requirement and an expectation, as a modern authority, to take advantage of all the technological innovations and opportunities available that could potentially create greater customer benefit.

“The stability of our facilities should be synchronized with digital development, which is not easy. In fear of losing stability, one does not want to shake up the current decision support. There is great potential in digitalization, but you do not want to risk doing wrong. You want control and stability in the first place.”

The respondents reported that new technologies could introduce new security risks such as data breaches, which could threaten the stability of the organization. Additionally, the introduction of new technologies can disrupt established processes and systems, requiring changes to be made in the way the organization operates, which may make it difficult for the organization to maintain control and stability.

### 4.4. Organizational Culture

The interviewees expressed a culture of what is called engineering thinking. By this, the interviewees meant that the technical expertise is high, but that the organizational and behavioral knowledge is given less space. “The Administration is a merger of different authorities with different cultures, and it is an extremely expert-heavy business with many specialized competences.”

The knowledge of change and business development was considered as too low. The culture of government also presupposes stability, which can lead to a reluctance to try new things. The security of employment and the absence of financial pressure seem to allow for working in old patterns without much focus on the “customer”. Incentives to change or their activities are described as low for those who have gained a position of power and there is no sense of urgency regarding the change. “We lack sense of urgency. No one here is worried about losing their job. Digitalization is the kind of change you resist. You wait and see.”

However, several of the interviewees expressed criticism that the culture did not benefit the challenges and complexity that existed in the business going forward. “We have a culture that is far from the end customer. How we deliver a service reflects that culture.”

“We need to talk about customer satisfaction and benefit and ask ourselves more questions about customers’ needs. But we don’t have the habit and the language for it, and we don’t have language for change.”

The respondents meant that engineers who had been working in a certain way for a long time may be hesitant to change their methods and embrace new technologies. Furthermore, they may be uncertain about how the new digital tools will impact their work and may be fearful of making mistakes or not understanding how to use the new technology. There may also be a lack of trust in new technologies, that the new technologies will not work as promised, or will not be able to handle the complexity of the work.

### 4.5. Lack of a Comprehensive View

The realization that systems understanding both within the Administration and outward to stakeholders would need to increase came up spontaneously in several interviews. The organizational structure was mentioned as a problem in the case of projects involving the entire Authority. “The Administration has a major flaw in systems thinking and overall understanding. We work in silos and the drivers of the different parts do not benefit the whole.

The desire for an authority encompassing process and transformation map was pointed out. The role of IT is described as reactive without a strong mandate due to the current organizational structure. “Unfortunately, we work in silos within the Administration. The IT landscape is also fragmented. The only area where we have accepted a common platform is salaries and communication. Otherwise, we have small fiefdoms. It has been difficult to transfer mandates from business areas to IT.”

Reward systems were perceived to encourage everyone to devote themselves to their part of the business. “We have a reward system that does not encourage a comprehensive approach and there is no reward or follow-up that is linked to the larger changes that we should make at the community level.”

Reflection on the challenge of obtaining a comprehensive view in such a diversified business was carried out, but several also stressed the importance of thinking more from the societal mission and the value that the Administration will create. Several expressed a belief in stronger management and governance toward the whole, better resource use, and cooperation. A large cultural displacement at work was mentioned as desirable.

Some examples of ongoing digitalization projects within the Administration are digital mapping and navigation, data analytics and reporting, and remote monitoring and maintenance. Digital mapping involves using digital technologies to improve the accuracy and functionality of road and transport systems, making it easier for drivers and passengers to find their way and plan their journeys. Data analytics and reporting involve using data analytics and visualization tools to gain insights from transportation data such as traffic patterns, usage, and customer behavior to improve the performance and efficiency of the transportation system. Remote monitoring and maintenance means using digital technologies such as sensors or remote monitoring to improve the maintenance, repair, and operation of the road and transport infrastructure.

## 5. Discussion

The aim of this study was to explore how employees within the Swedish Transport Administration experienced change efforts focused on digitalization and change within the Swedish Transport Administration. The results showed that there was a lack of a comprehensive view on digitalization, what it means, and how it can support the mission of the Administration.

The digitization process that the Swedish Transport Administration is currently undergoing, with several ongoing digitization initiatives and newly implemented roles, is a transformative change that will change both the structure and working methods within the authority and also affect the culture and power relations [50]. Such a change will most likely lead to a reconsideration of old ways of working and truths [51,52]. In order for such a change to succeed, there needs to be a clear path of the transformation and support for local change agents [37,53]. The results in this study point toward a lack of understanding among first-level management for the current situation. Strategists and local change agents trying to manage change have experienced that change and transformation is not prioritized or supported. Such sponsorship is a necessary prerequisite for successful transformational change [54].

Transformative changes take time to implement [13] and they need to be clearly communicated by management and be anchored in all parts of the organization in order to achieve success [19]. This was supported by the results of the present study where the Administration clearly lacks a management structure that can handle both change and innovation. Such management control is sometimes referred to as ambidextrous organizations [55]. Outside pressures are likely to enhance the conflict between predictable administration and development and innovation. This pressure, and the imbalance between internal organizational structures and the experience needed for innovation and change, are reported in the findings of the present study.

Norrman Brandt et al. [13,56] showed how a leadership characterized by a delegated decision-making mandate, emotional support, and a comprehensive and cohesive view of the organization and change was successful in major transformative organizational changes. Such an alternative leadership created the conditions for implementing major, comprehensive changes to an organization. Other studies have shown that modern organizations need to develop new work processes to meet the changes that digitization brings. Examples of new areas that are highlighted are control and management via digital systems, increased knowledge and skills regarding technical systems among both employees and customers, an increased use and analysis of data, and an increased general ability to innovate in the organization [57].

The results indicate that the organizational readiness for change [58] is low within the Administration. Change effectiveness tends to be higher if there is a collective self-confidence and readiness to act [59]. Consistent communication from all leaders in an organization as well as the opportunity to meet and talk across different parts of the organization also favors readiness for change [56]. This is clearly not the present situation within the Transport Administration, according to the results of the study.

### 5.1. Limitations

This study was limited to statements from respondents in an interview situation. Such statements cannot always be taken as empirical facts of a real situation [60]. Another limitation of the present study is the fact that a limited number of interviews were conducted. Forthcoming research will be focused on how change comes about and how the process of change develops over time in a large national Administration. This would be in line with the call from [40], who noted the present lack of process-oriented studies in the theoretical research field of change and change management.

### 5.2. Conclusions

The results of this study indicate that the Transport Administration, a large Swedish governmental agency, still has a long way to travel in terms of organizational readiness for change. If this is the case in one large governmental agency, it is reasonable to believe that other agencies also need to catch up in this regard. Readiness for change needs to be elevated at all levels of the organization and the stability requirement needs to be challenged. In order to address this issue, the Transport Administration should prioritize the development and implementation of a comprehensive change management strategy. This should include clear communication, active engagement and participation from all employees, and a focus on building a culture of adaptability and continuous improvement, which will allow it to navigate future changes more easily and effectively. Additionally, senior management should lead by example and actively work to create a sense of urgency and momentum for change. Thus, the Transport Administration needs to take action in order to improve its organizational readiness for change. The agency should focus on a change management strategy that addresses the needs of all employees, and actively engage with staff at all levels to ensure that they understand the reasons for change and how it will benefit them. By taking these steps, the Transport Administration can improve its organizational readiness for change and set a positive example for other governmental agencies to follow, and better serve the needs of the public it serves.

## Data Availability

Not applicable.
